# Probing the orbital angular momentum of intense vortex pulses with strong-field ionization

**DOI:** 10.1038/s41377-022-00726-7

**Published:** 2022-02-08

**Authors:** Yiqi Fang, Zhenning Guo, Peipei Ge, Yankun Dou, Yongkai Deng, Qihuang Gong, Yunquan Liu

**Affiliations:** 1 State Key Laboratory for Mesoscopic Physics and Frontiers Science Center for Nano-optoelectronics, School of Physics, Peking University, Beijing, 100871 China; 2 Collaborative Innovation Center of Extreme Optics, Shanxi University, Taiyuan, Shanxi 030006 China; 3 Center for Applied Physics and Technology, HEDPS, Peking University, Beijing, 100871 China; 4 Beijing Academy of Quantum Information Sciences, Beijing, 100193 China

**Keywords:** Nonlinear optics, Optical techniques

## Abstract

With the rapid development of femtosecond lasers, the generation and application of optical vortices have been extended to the regime of intense-light-matter interaction. The characterization of the orbital angular momentum (OAM) of intense vortex pulses is very critical. Here, we propose and demonstrate a novel photoelectron-based scheme that can in situ distinguish the OAM of the focused intense femtosecond optical vortices without the modification of light helical phase. We employ two-color co-rotating intense circular fields in the strong-field photoionization experiment, in which one color light field is a plane wave serving as the probing pulses and the other one is the vortex pulses whose OAM needs to be characterized. We show that by controlling the spatial profile of the probing pulses, the OAM of the vortex pulses can be clearly identified by measuring the corresponding photoelectron momentum distributions or angle-resolved yields. This work provides a novel in situ detection scenario for the light pulse vorticity and has implications for the studies of ultrafast and intense complex light fields with optical OAM.

## Introduction

Optical vortex beams carry the well-known optical orbital angular momentum (OAM) of *ℓћ* per photon^1^, where *ℓ* is usually an integer number and denotes the topological charge of the field. Optical OAM provides a very important degree of freedom for a variety of fields in modern science, such as particle tweezing^2^, quantum communication^3^, telecommunications^4^, astrophysics^5^, microscopy^6^, and biology^7,8^. Since the dynamic signature of vortex beams is determined by the OAM mode (or the topological charge)^9^, the OAM mode measurement is one of the crucial tasks which is prior to the applications of vortex beams. Hitherto, the OAM detection schemes are mostly achieved by optical methods. One main approach is to utilize the interferometric techniques, in which the number of the stripes in the specific interferograms are related to the topological charges^10–13^. Another traditional choice is to use diffraction patterns with specific masks, such as triangular aperture diffraction^14^, annular aperture diffraction^15^, and linear or angular slit(s) diffraction^16,17^. In addition, some methods involving such as conformal mappings^18,19^, multiplane light conversion^20^, robust mode converter^21^, rotational Doppler effect^22^, and two-dimensional material^23^, have also shown capabilities in measuring the OAM of vortex beams.

Almost all of these traditional optical methods can be regarded as a strong coupling detection, and the measurement itself will inevitably modify the OAM state of the light beam. To overcome this problem, recently, the weak measurement of optical OAM has been both theoretically^24^ and experimentally^25^ demonstrated. The weak measurement typically involves three stages^26^: the system to be measured is prepared in initial states; then the system weakly interacts with a probe; at last, the final state of the system is post-selected. However, for the complicated weak measurement method, it is also hard to achieve the non-destructive detection of photon OAM. Thus, it is worthwhile to seek alternative and straightforward methods to probe light OAM state without the OAM state collapse, so that the measured optical vortex beams can then be utilized in subsequent application.

In strong-field community, benefited from the rapid development of ultrafast lasers, the femtosecond vortex pulses have attracted growing attention in the study of intense-light-matter interaction. Today, the vortex pulses have been used in ultrafast spatio-temporal manipulation on the topological fields in the extreme ultraviolet (EUV) region, such as generating the EUV beam with time-varying OAM^27^ and simultaneous spin–orbit momentum control on the attosecond pulses^28^. The intense vortex pulses also give rise to new selection rules for photoionization or photoexcitation process, accompanied by unique angular momentum transitions^29,30^. Recently, the OAM-dependent dichroic photoelectric effect^31^ and the spin–orbit coupling of intense light fields in optical focusing systems^32^ were successively demonstrated via photoionization experiments. In strong-field regime, the interaction between the intense laser pulses and targets is usually performed in a high-vacuum circumstance, the light pulses have to be focused to a small spatial scale to achieve high intensity, and the highly concentrated laser energy is destructive for optical instruments. These obstacles hinder the applicability of the conventional optical methods in probing the OAM of strong-field vortex pulses in situ. Up to now, the measurement and application of light OAM have been less studied in those intense-light-matter interaction processes.

Here we propose and demonstrate an approach for in situ probing the OAM of intense vortex pulses with photoelectron momentum imaging via strong-field photoionization experiment. In the experiment, the ionization rate is controlled under the condition that less than one atom is ionized per laser pulse. In such intense laser pulse, there are a large number of photons (>10^15^) and only about 10 photons have been absorbed in multiphoton ionization of Argon atom, and thus the measurement process has almost no influence on the OAM states of vortex pulses. Optically, we employ a synthesized two-color co-rotating circular laser field configuration, in which a plane wave is used as a probing light to detect the helical phase structure of the other vortex pulses. The core of this method is to link the relative-phase structure of two-color light to the photoelectron momentum distributions or angle-resolved yields. In the experiment, we successfully characterize the high-power femtosecond vortex pulses carrying three different OAM modes (*ℓ* = 0, 1, and 2). We support this scheme with the simulations of the semi-classical models. Basing on the experimental observation and theoretical prediction, we further propose a universal scheme for the detection of strong-field vortex pulses with higher OAM modes.

## Results

### Experimental configuration and focal light field distributions

**Fig. 1:**
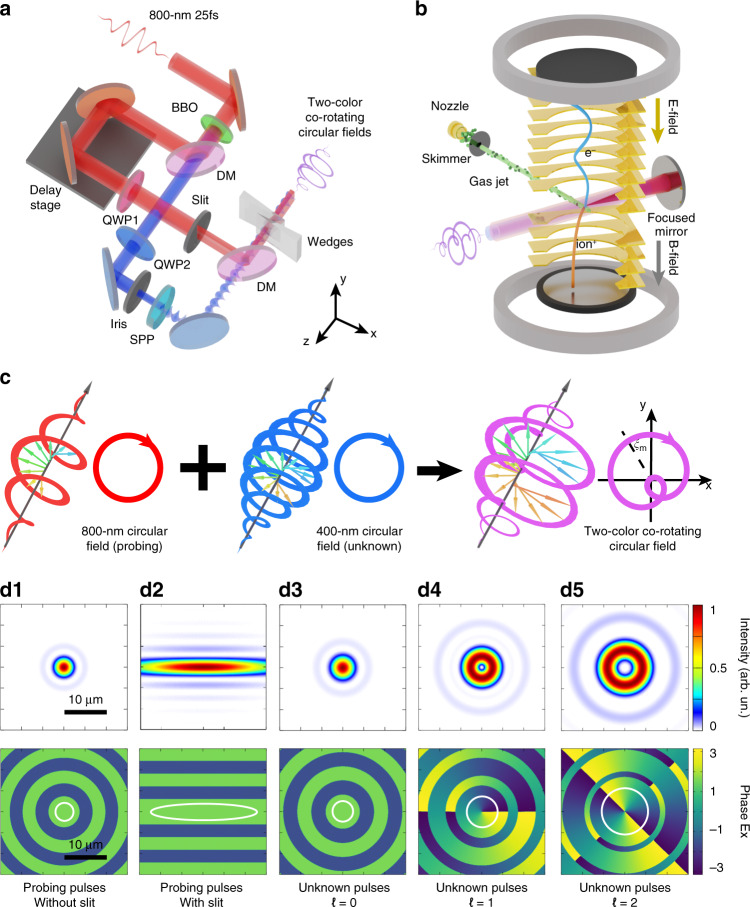
Schematic of the experiment. **a** Layout showing the preparation of the two-color co-rotating circular light fields with the 800-nm light pulses (red) and 400-nm light pulses (blue). A Mach–Zehnder interferometer scheme is used. BBO, barium borate crystal; DM, dichroic mirror; QWP, quarter-wave plate; SPP, spiral phase plate. **b** Illustration of the strong-field ionization detection experiment. The generated two-color co-rotating circular field interacts with the supersonic argon atom gas jet inside of COLTRIMS apparatus. **c** Diagram of the electric field vectors of the 800-nm circular field (red), 400-nm circular field (blue), and two-color co-rotating circular field (purple), respectively. For each laser field, a 3D polarization profile (left) and a 2D Lissajous figure of its electric field in transverse plane (right) are given. The deflected angle of peak electric field of the two-color field, *ξ*_m_, is related with that in Fig. 2a. **d1**–**d5** The simulated light spatial profiles in the focal plane. The top panels show the normalized intensity distributions and the bottom panels show the phase structure of *x*-component electric field. The white circles enclose the regions (exclude the singularities in b4 and b5), where the light intensity is larger than 0.5*I*_m_ (*I*_m_, the peak intensity of focal light field)

We illustrate the experimental scheme in Fig. 1a, b. The fundamental laser pulses (800-nm, 25 fs) are delivered from a multipass Ti:sapphire laser amplifier operating at 3 kHz. And the second harmonic (400-nm) is produced through the frequency doubling with a 250-μm-thick *β*-barium borate (*β*-BBO) crystal. In the experiment, we use the 800-nm light field as the probing pulses and the 400-nm light field whose OAM needs to be characterized as unknown pulses. The probing pulses and the unknown pulses are individually controlled by a Mach–Zehnder interferometer scheme. In the arm of probing pulses, a home-made 2-mm horizontal slit can be optionally used to sculpt the spatial profile of light beam. In the arm of unknown pulses, an iris aperture is utilized to control the spatial scale of the unknown pulses at focus. We transduce the light into vortex pulses by using spiral phase plates^33^. Here, we prepare the unknown beams with three different OAM modes (*ℓ* = 0, 1, and 2). In the experiment, the beam radii of probing pulses and unknown pulses were controlled to be ~9 and ~4.5 mm (FWHM), respectively. As shown in Fig. 1c, the polarizations of the probing pulses and unknown pulses are both adjusted to be right-circularly polarized, and their synthesized laser field is called two-color co-rotating circular laser field. The relative time delay Δ*t* between the probing beam and the unknown beam is finely tuned by a pair of fused silica wedges with a precision of roughly 0.004π rad (~5 attosecond).

Then, as shown in Fig. 1b, we focus the two-color co-rotating circular fields by a 75 mm focal length mirror (numerical aperture = 0.17) into the high-vacuum chamber (5 × 10^−11^ mbar) of the cold-target recoil-ion-momentum spectroscopy (COLTRIMS) set-up^34^. In the laser-atom interaction region, the peak intensities of the probing pulses and unknown pulses are calibrated to be *I*_pr_ = 5.6 × 10^13 ^W cm^−2^ and *I*_un_ = 3.5 × 10^12 ^W cm^−2^, respectively^35^. At the focus, the synthesized field interacts with the supersonic argon atom gas jet. The generated photoelectrons are measured by a time-of-flight spectrometer with a position-sensitive detector, and their three-dimensional photoelectron momentum distributions are then constructed (see “Methods” for more details).

To analyze the geometry of the two-color light field in focal plane, we simulate the spatial distributions of light fields by using the Richards–Wolf vectorial diffraction method^36^. In Fig. 1d, we present the calculated distributions of the intensity and phase of *x*-component electric field. The intensity of the probing pulses reveals as a Gaussian-like distribution in focal plane (Fig. 1d1). Note that when the 2-mm horizontal slit is added in the light path, the intensity distribution of the probing pulses at the focus is stretched horizontally (Fig. 1d2). Here, we refer to the probing pulses without the slit (Fig. 1d1) and with the slit (Fig. 1d2) as the non-spatially sculpted and spatially sculpted probing pulses, respectively. On the other hand, the spatial size of unknown pulses is related to the OAM value (Fig. 1d3–d5). And the focal distribution of laser intensity manifests as the typical donut-like structures (Fig. 1d4, d5). The simulated results show that in the focal plane, the spot of unknown pulses (Fig. 1d3–d5) is larger than the spot of non-spatially sculpted probing pulses (Fig. 1d1). Such light configuration results in an important consequence that the horizontal size of the spatially sculpted probing pulses is much larger than the spots of unknown pulses, but meanwhile, its vertical size is smaller than the probing pulses. As shown later, this will allow one to subtly probe the topological phase structure of the unknown pulses experimentally.

### Probing the OAM with non-spatially sculpted probing pulses

**Fig. 2:**
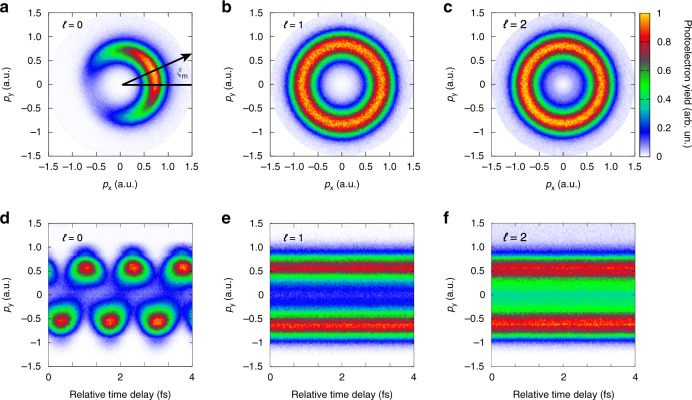
Probing the OAM of intense vortex pulses with the non-spatially sculpted probing pulses in strong-field ionization measurement. **a**–**c** Measured photoelectron momentum distributions by the two-color fields. **d**–**f** The sub-cycle time-dependent momentum distributions. The topological charges of the unknown light fields are (**a**, **d**) *ℓ* = 0, (**b**, **e**) *ℓ* = 1, and (**c**, **f**) *ℓ* = 2, respectively

First, we use the non-spatially sculpted probing light field (Fig. 1d1) to probe the OAM of unknown pulses (Fig. 1d3–d5). We stabilize the relative time delay of the two-color field (Δ*t* is constant), by fixing the position of the wedges in the experiment. Then, we measure the photoelectron momentum distributions (PMDs) by employing the unknown pulses of different topological charges in the two-color laser field. It can be observed that when *ℓ* is zero, the PMD reveals as a typical crescent-shaped lobe (Fig. 2a). By contrast, when *ℓ* is non-zero, the PMDs manifest as perfect annulus structure (Fig. 2b, c).

Such prominent phenomenon is intrinsically associated with the relative phase Δ*ϕ* of synthesized two-color field. In temporal domain, the Lissajous curve drawn by the tip of the co-rotating electric field vector has a single maximum per laser cycle. The ionized photoelectrons by this maximum in the periodically electric field form a peak yield in electron momentum distribution, leading to a crescent-shaped lobe^37^. One should note that the deflected angle *ξ*_*m*_ of the crescent-shaped lobe is one-to-one correlated with the relative phase Δ*ϕ*. And the relation can be given by: *ξ*_*m*_ = −Δ*ϕ* (see “Methods” for more details). Here, the relative phase is determined by the relative time delay Δ*t* and the spatial phase structure of two-color field, i.e., Δ*ϕ* = *ω*_un_Δ*t* + *ℓφ*, where *ω*_un_ is the angular frequency of the unknown pulse and *φ* = tan^−1^(*y*/*x*) is the azimuthal angle in focal plane. Since the photoionization can occur at any point in the focal light spot, the measured PMD on the “detector” is the superposition of the photoelectron signals produced from the whole focal plane. When the OAM of the unknown pulses is zero, the relative phase of the two-color field is spatially uniform. The photoelectrons ionized from different positions in the focus have PMDs with the same deflected angle, thus revealing the typical crescent-shaped lobe in Fig. 2a.

**Fig. 3:**
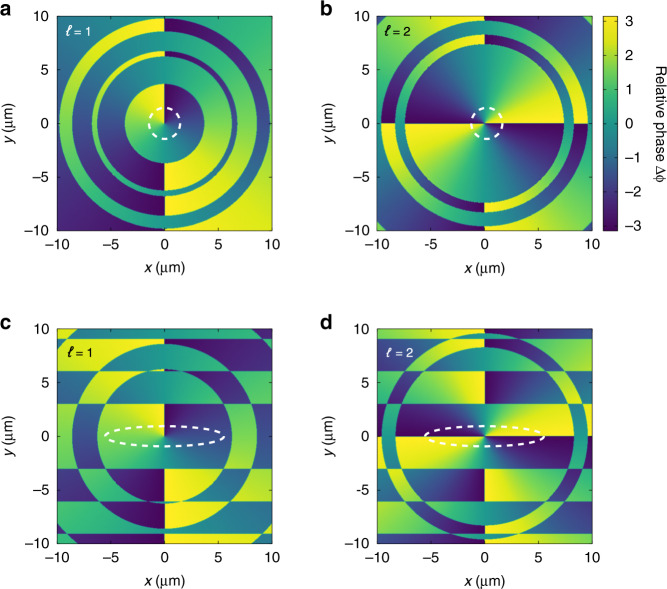
Relative phase distributions of the two-color light fields in focal plane. **a**, **b** The spatial structure of the two-color field is not sculptured. **c**, **d** The probing light in the two-color field is sculptured by a 2-mm horizontal slit. The OAM modes of the unknown fields are *ℓ* = 1 (**a**, **c**) and *ℓ* = 2 (**b**, **d**). The dashed lines encircle the regions where the photoionization rate is dominated

When the unknown fields possess non-zero OAM, the relative phase distributions of two-color field are not uniform in focal plane (Fig. 3a, b), which is different from the case of *ℓ* = 0. We calculate the local photoionization rate distributions by using the Ammosov–Delone–Krainov (ADK) theory^38^, and delineate the dominated region (see dashed circles in Fig. 3) where the photoionization rate *W*(*x*,*y*) is larger than 0.05*W*_m_ (*W*_m_ is the maximal local photoionization rate in focal plane). As shown in Fig. 3a, b, the relative phase of two-color field is azimuthal-dependent in this dominated region. The photoelectrons released from different azimuths in focal plane form PMDs with different deflected angles. These deflected angles range from 0 to 2*πℓ*, and thus, the measured PMD on the “detector” reveals as an annulus (Fig. 2b, c).

In the experiment, we further scan the relative time delay of two-color fields by moving the wedges, and the PMDs are recorded simultaneously (Fig. 2d, e). For *ℓ* = 0, the photoelectron momentums along *p*_*y*_-direction will oscillate with respect to the time delay (Fig. 2d). As for *ℓ* = 1 and *ℓ* = 2, the notable difference is there will be no oscillation anymore (Fig. 2e, f). Here, the oscillation disappears because both the spatial phase structure of the unknown pulse and the time delay of two-color field come into effect in modulating the sub-cycle momentum of photoelectrons. The experimental results well indicate that the presence of intense laser pulse OAM can be identified by monitoring either PMDs or sub-cycle time-dependent momentum distributions.

One can notice that the difference between *ℓ* = 1 and *ℓ* = 2 is not significant. Essentially, the indiscernibility between these two OAM modes stems from the spatial cylindrical symmetry of the synthesized two-color laser field. Such a symmetrical distribution leads to the fact that the photoelectrons released from different azimuthal angles in the focal light spot contribute equally to the measured PMD. Thus, although the winding number of phase structure is different for *ℓ* = 1 and *ℓ* = 2 (see Fig. 3a, b), it is not recorded by the photoionization observables.

### Probing the OAM with spatially sculpted probing pulses

In order to visualize the more detailed phase structures of vortex pulses, the key is to control the morphology of two-color laser fields and redistribute the weights of the released photoelectrons in the focal light spots. To this end, we use a 2-mm horizontal slit in the probing light path so that the cylindrical symmetry of the probing field can be broken as well as the synthesized two-color laser field.

In this scenario, when the spatially sculpted probing light field (Fig. 1d2) overlaps with the donut-shaped unknown pulses (Fig. 1d4, d5), the intensity distribution of the synthesized field is stretched horizontally. The dominated photoionization region is also stretched (dashed circles in Fig. 3c, d), so that when employing such two-color laser fields in strong-field ionization measurement, most of the photoelectrons are released from the local relative phase along the line *y* = 0 in the focal plane (labeled by Δ*ϕ*|_*y*=0_). For *ℓ* = 1 (Fig. 3c), we have Δ*ϕ*|_*y*=0_ = −*π*/2, and *π*/2. However, for *ℓ* = 2 (Fig. 3d), it becomes Δ*ϕ*|_*y*=0_ = −*π* and *π*. Such diversity in the phase distributions of the two-color laser fields originates from the different parities of the optical OAM between *ℓ* = 1 and *ℓ* = 2. Note that the crescent-shaped lobes in PMDs have opposite deflected angles for Δ*ϕ*|_*y*=0_ = −*π*/2 and *π*/2. But the crescent-shaped lobes driven by Δ*ϕ*|_*y*=0_ = −*π*, and *π* have the same deflected angle.

**Fig. 4:**
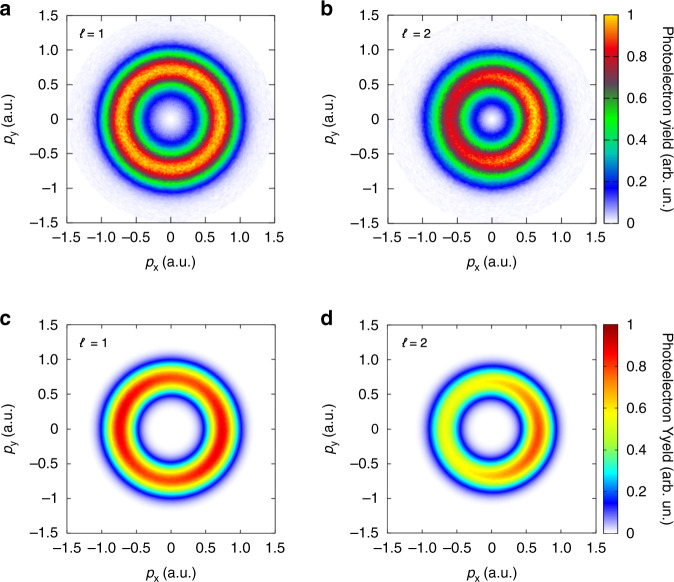
Probing the OAM of intense vortex pulses with the spatially sculpted light field. **a**, **b** Measured photoelectron momentum distributions. **c**, **d** Simulated photoelectron momentum distributions. The topological charges of 400-nm fields are (**a**, **c**) *ℓ* = 1 and (**b**, **d**) *ℓ* = 2, respectively

Experimentally, we measure the PMDs by using the above spatially sculpted two-color laser fields. As shown in Fig. 4a, b, the PMD for *ℓ* = 1 is still an annulus, but the PMD for *ℓ* = 2 will reveal a crescent-shaped lobe. Such two quite different PMDs provide the observables for the detection of light pulse OAM. Here, the generation mechanism of the annulus in Fig. 4a is partly different from the annuli in Fig. 2b, c. Since the photoelectrons ionized by Δ*ϕ*|_*y*=0_ = −*π*/2 and Δ*ϕ*|_*y*=0_ = *π*/2 form two crescent-shaped lobes with opposite deflected angles, the maximal yield in one lobe exactly compensates for the minimal yield in the other lobe. Hence, the measured total PMD still looks like an annulus (Fig. 4a).

In this experiment, since the OAM information of the vortex field is recorded by the overlap region of these two laser pulses, most of the photoelectrons are released from the overlap region. Here, if the slit width is too larger, the horizontal size of the focused probing pulse will be much smaller. Once the focal horizontal size of the probing pulse is shorter than the radial distance of the donut shape of the unknown pulse, the yield of photoelectrons released from the overlap region of two light fields will be decreased, and thus the OAM probing precision could be affected.

To further support the detection scheme, we have performed the simulation with the classical-trajectory Monte Carlo (CTMC) model^39,40^, in which we have fully considered the OAM-induced spatial intensity structure and helical phase structure of the two-color laser field (see “Methods” for more details). In simulation, the photoelectrons released from different points in focal plane are incoherently superposed in momentum space. As shown in Fig. 4c, d, the simulated results are in good agreement with the experimental observation. Hence, the OAM of unknown pulses with *ℓ* = 1 and *ℓ* = 2 can be clearly characterized by controlling the profile of probing light. Furthermore, we theoretically show that the chirality of probing beams’ OAM and spin angular momentum (SAM) provides a robust toolbox for the measurement of the OAM of optical vortices (Supplementary Information).

### Universal scheme for probing the OAM of intense vortex pulses

To probe the high-order OAM of intense vortex pulses using strong-field ionization, we derive the angle-resolved photoelectron yield *W*(*ξ*,*φ*) (see “Methods” for more derivation details), where *ξ* = tan^−1^(*p*_*y*_/*p*_*x*_) is the electron emitting angle in momentum space, and *φ* denotes the azimuthal angle in the focal plane where the photoelectron is ionized. Mathematically, *W*(*ξ*,*φ*) is given by

1 $$W(\xi ,\varphi ) = [(2I_p)^2/\left| {E(\xi ,\varphi )} \right|]^{2/\sqrt {2I_p} - 1} \cdot \exp \left( - \frac{{2(2I_p)^{3/2}}}{{3\left| {E(\xi ,\varphi )} \right|}}\right)$$

where *I*_*p*_ is the ionization potential for the model argon atom (*I*_*p*_ = 15.8 eV) and $\left| E \right| = \sqrt {\varepsilon _{{{{\mathrm{pr}}}}}^2g_{{{{\mathrm{pr}}}}}^2 + \varepsilon _{{{{\mathrm{un}}}}}^2g_{{{{\mathrm{un}}}}}^2 + 2\varepsilon _{{{{\mathrm{pr}}}}}\varepsilon _{{{{\mathrm{un}}}}}g_{{{{\mathrm{pr}}}}}g_{{{{\mathrm{un}}}}}\cos (\xi + \Delta \phi )}$ is the instantaneous electric field strength, in which the ionization instant *t*_0_ has been replaced by the mapping relation *t*_0_ = *ξ*/*ω*_pr_ (see “Methods” for details). The subscript “pr” and “un” describe the parameters of probing pulses and unknown pulses, respectively. *ɛ* is the peak electric field strengths, *ω* is the angular frequency of probing pulses, *g*(*φ*) is the normalized azimuthal intensity profile, and Δ*ϕ* = *ω*_un_Δ*t* + *ℓφ* is the relative phase of the two-color field. It is worth noting that we can interpret the photoelectron yields in the above experimental results (Figs. 2a, b, c, 4a, b) by using this analytical model (“Methods”).

**Fig. 5:**
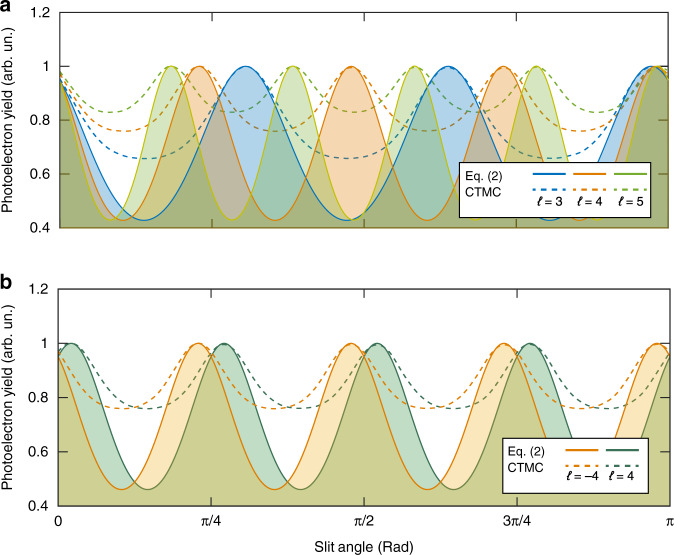
Universal scheme for detecting light OAM by employing strong-field ionization measurement. We inspect the yields of photoelectrons, whose emitting angles *ξ* are confined in a range (*ξ* ϵ [0, π/6]), with respect to the slit angle. Different topological charges of probing light fields (*ℓ* = 3, ±4, and 5) can be clearly revealed according to the peak number and position of the photoelectron yields. The solid lines illustrate the results of our analytical model and the dashed lines show the calculations by using the CTMC model. **a** The results for OAM modes of different magnitudes (*ℓ* = 3, 4, and 5). **b** The results for OAM modes of opposite signs (ℓ = ±4)

In Eq. (1), *W* is correlated with the instantaneous electric field strength |*E*|. Here, |*E*| is connected with *ξ*, Δ*t*, and *ℓφ*. This suggests that if one can control *ξ* and Δ*t* to be fixed, the topological charge of light pulses is included in the relationship between *W* and *φ*. Technically, controlling *ξ* and Δ*t* is not hard. Besides, as shown in Fig. 3c, d, one can also realize the control over *φ* by employing a slit. Based on these, we propose a universal scheme for detecting the higher OAM modes of intense vortex pulses. Here, one continuously rotates the slit experimentally so that the azimuthal position of the photoionization events can be well controlled. In the meantime, one just needs to measure the photoelectrons yields, whose emitting angles are confined in an angle interval, e.g., *ξ* ϵ [0, π/6]. Theoretically, the relationship between such photoelectron yield *Wʹ* and the slit angle *φ*_s_ can be written as:

2 $$W^\prime (\varphi _s) = {\int_0^{\pi /6}} {W(\xi ,\varphi _s) + W(\xi ,\varphi _s + \pi )} d\xi$$

in which the effect of slit has been mathematically approximated as *g*(*φ*) = *δ*(*φ* − *φ*_s_) + *δ*(*φ* − *φ*_s_ − *π*), where $$\delta (x) = \left\{ {\begin{array}{*{20}{c}} {1\quad (x = 0)} \\ {0\quad (x\, \ne \,0)} \end{array}} \right.$$. Without loss of generality, here we use the 400-nm plane wave as the probing pulses and the 800-nm vortex pulses as the unknown pulses carrying various OAMs. We calculate *Wʹ*(*φ*_s_) by using Eq. (2). As shown in Fig. 5a, when varying the angle of slit, there are different numbers of maximums of *Wʹ* for different OAM values. It is worth noting that the number of maximums is exactly equal to the value of topological charge. Moreover, the phase of extremums can clearly reflect the sign of the OAM value as shown in Fig. 5b (solid lines). To support the scheme, we also perform the simulation with the CTMC model (dashed lines in Fig. 5a, b). As shown, the results from the CTMC model show good agreement with the analytical method (Eq. (2)). Especially, the locations of the extremums calculated by these two methods have an excellent agreement. Here, one may find that there is a deviation in the contrast between the maxima and minima yield of the results from these two models. In the analytical method, the initial momentum of electrons at the tunnel exit is discarded, but in the CTMC model, the initial transverse momentum is chosen to be a Gaussian distribution centered at zero. Thus, as shown in Fig. 5, the contrast between the maxima and minima yield obtained by the CTMC model is smaller than that obtained by the analytical model.

## Discussion

In photoionization, because of the large difference between the focal spot of light and the electron ponderomotive motion in the intense light field, the electrons cannot “see” the global geometric structure of the driving laser field^41^. So, it is hard to directly embody optical OAM in the dynamics of electrons in photoionization process. To overcome this difficulty, here we have used the synthesized two-color co-rotating circular laser field configuration. In this geometry, the effect of optical OAM in strong-field ionization can be clearly revealed. It is worth noting that the photoionization signal is also sensitive to the polarization state of interacting laser fields. Hence, this method has the potential to further characterize more complex laser fields, such as vector light fields and even more complicated optical skyrmionic beams^42^.

The photoionization method presented in this work is fundamentally different from the traditional optical methods. The photoelectron momentum distributions and yields can directly record the information of spatial phase structure of vortex pulses. In our experiment, we have controlled each light pulse to ionize only a single atom. The first ionization potential of argon atom is *I*_p_ = 15.8 eV and the most parable emitting energy of ionized electron *E*_k_ is ~7 eV (Fig. 2). The photon energy of 800-nm and 400-nm light field is *ω*_800_ = 1.55 eV and *ω*_400_ = 3.1 eV, respectively. We assume that the numbers of absorbed 800-nm photon and 400-nm photons are *n*_800_ and *n*_400_. According to energy conservation, then we will have: *n*_800_*ω*_800_ + *n*_400_*ω*_400_ = *I*_p_ + *E*_k_. Though there are different absorbed photon channels (*n*_800_, *n*_400_), we can well speculate that the number of photons absorbed from the laser pulses is approximately *n*_800_ + *n*_400_ ~ 10, which is very trivial compared to the total number of photons per laser pulse (>10^15^). That is, the probe process has almost no effect on the laser OAM state. Such unique feature makes the photoionization method an ideal and unique tool for measuring the quantum states of optical systems, such as the OAM or spin angular momentum of photons. The measured optical systems can be used in further broad applications.

In conclusion, we have demonstrated a novel detection scenario of OAM of high-intensity laser pulses, on the basis of the strong-field ionization process. A synthesized two-color co-rotating circular laser field configuration was utilized in this scheme, in which a plane wave is used as a probing light to visualize the OAM of the other vortex pulses. To support this scenario, we have presented the experimental and theoretical results. Currently, this method might be the only sufficient method for the in situ detection of light pulse OAM in intense-light-matter interaction. The methodology used in this work can be extended to the control of EUV radiations in high-order harmonic generation using the intense vortex pulses^27,28,43,44^, which may largely facilitate the spatio-temporal control over the radiation of high-energy photons. Moreover, this work has also implications for generating and probing the structured electron beams, such as electron vortices^45,46^.

## Materials and methods

### Experimental details

The *p*-polarization laser pulses were delivered from a multipass Ti:sapphire laser amplifier at a central wavelength of 800-nm. The phase structure of the 400-nm light pulse was imprinted by the commercial spiral phase plates whose topological charges were 1 and 2, respectively. The spiral phase plates were mounted on a two-dimensional motorized linear stage. Before illuminating the 400-nm vortex pulses into the COLTRIMS set-up, the spatial intensity distributions of the pulses were monitored by a Charge Coupled Device (CCD) camera, ensuring that the phase singularities of the generated vortex pulses were located at the beam centers. To accurately control the relative time delay of the two-color fields, the wedges with an angle of 5° were mounted on a one-dimensional motorized linear state, whose minimum achievable incremental movement was 0.05 μm. The 800-nm and 400-nm light pulses were focused by a silver-coated concave mirror with focal length *f* = 75 mm, which was placed inside the high-vacuum chamber (5 × 10^−11^ mbar) of the COLTRIMS set-up. In the high-vacuum chamber, the spatial alinement of the 800-nm and 400-nm light fields was monitored by the photoionization counts and finely controlled by rotating the two rotary knobs on the optical frames in the 800-nm light path. The polarization and intensity of the light fields were jointly controlled by wire grid polarizers and *λ*/2 and *λ*/4 retardation plates. In order to obtain a clear crescent-shape lobe in photoelectron momentum distribution, we control the intensity of 800-nm light field (i.e., the probing field) being larger than the 400-nm light field (i.e., the optical vortex field)^47^. Here, if the helicity of 800-nm light field is opposite to the helicity of 400-nm light field, the synthesized two-color field is called counter-rotating two-color circular field. Such light field is also workable in our OAM probing scheme. Since there are three lobs in the electron momentum distribution when using the counter-rotating field^37^, such laser field demands a much higher resolution of the experimental results. Hence, we selected the co-rotating field in our experiment.

In our COLTRIMS, the supersonic gas jet of argon atoms was delivered along the *x*-direction by a small nozzle ~30 μm. Then, a 290 μm skimmer was used to filter the gas jet and to monitor the gas pressure of the main chamber. After that, a pair of small holes (diameter 1 mm) was used to collimate the supersonic gas jet, so that the transverse motion of the gas atoms can be mitigated. Noted that the focal spot of the light field (~10 μm) was much smaller than the size of the gas jet (~1 mm). In the interaction regime of the laser with the argon atoms, the electrons and ions produced by the strong-field ionization were guided to the two-dimensional position-sensitive detectors by a homogeneous electric field (~3.3 V cm^–1^) and a weak homogeneous magnetic field (~6.5 G) along the time-of-flight axis. The electric field was used to accelerate and collect the electrons and ions. The magnetic field was used to compensate for the Earth’s magnetic field and to guide the electrons to achieve all solid angle measurement. The momenta of electrons and ions are measured with the position-sensitive detectors with microchannel plates equipped with delay lines. The electrons and their parent ions were measured in coincidence to avoid the false signals of electrons.

### Focal light field simulation

As shown in Fig. 1a, the light fields propagate along *z*-direction. Here, the temporal envelope of pulse was neglected in the simulation of focal light fields (Figs. 1d and 3). The incident light fields in the plane of the focused mirror can be approximately expressed as: **E**_i_(*xʹ*,*yʹ*) = *D*(*xʹ*,*yʹ*)*e*^i*ℓ*atan(*yʹ*/*xʹ*)^*H*(*d*/2-|*xʹ*|), where the symbols with (′) indicate the transverse coordinates in the focused mirror plane, *D* is the amplitude distribution in the polarization plane, *H* represents a Heaviside function denoting the effect of the slit, and *d* is the slit width.

To accurately reproduce the electric fields at the focal plane, we employed a non-paraxial diffraction method, that is, the Richards–Wolf vectorial diffraction method^36^, to calculate the field near the focus **r** = (*x*, *y*, *z* = 0):

3 $$\begin{array}{ll}{{{\mathbf{E}}}}_{{{\mathrm{f}}}}({{{\mathbf{r}}}}) = \frac{{ - ikf}}{{2\pi }}\mathop {\int}\limits_0^{\theta ^\prime _m} {\mathop {\int}\limits_0^{2\pi } {{{{\mathbf{E}}}}_i(x^\prime ,y^\prime )P(\theta ^\prime )} } \cdot \exp (ik[z\cos \theta ^\prime + \rho \sin \theta ^\prime \cos (\varphi ^\prime - \varphi )])\\ \qquad \qquad\times \left[ {\zeta ^\prime \left( {\begin{array}{*{20}{c}} {\cos \theta ^\prime \cos (\varphi ^\prime - \varphi ){{{\mathbf{e}}}}_\rho } \\ {\cos \theta {^\prime}\sin (\varphi {^\prime} - \varphi ){{{\mathbf{e}}}}_\varphi } \\ { - \sin \theta ^\prime {{{\mathbf{e}}}}_z} \end{array}} \right) + \gamma ^\prime \left( {\begin{array}{*{20}{c}} { - \sin (\varphi {^\prime} - \varphi ){{{\mathbf{e}}}}_\rho } \\ {\cos (\varphi {^\prime} - \varphi ){{{\mathbf{e}}}}_\varphi } \\ 0 \end{array}} \right)} \right]\sin \theta ^\prime d\varphi ^\prime d\theta ^\prime \end{array}$$

where *f* is the focal length of the mirror, *ρ* = (*x*^2^ + *y*^2^)^1/2^ and *φ* = atan(*y*/*x*) are the radial distance and the azimuthal angle in the focal plane respectively, *φ*′ = atan(*yʹ*/*xʹ*) is the azimuthal angle in mirror plane, *ζʹ* and *γʹ* are the radial and azimuthal amplitude factors respectively, *k* = 2*π*/*λ* is the wavenumber, *θʹ* is the polar angle in the output pupil of the focusing system, *θ*_m_′ is the maximal angle determined by the numerical aperture of mirror, and *P*(*θ*′) = (cos*θ*′)^1/2^ is the apodization factor. In the simulations, the circularly polarized fields are used, and thus the radial and azimuthal factors are *ζʹ* = sin(*φ*′) + cos(*φ*′)*e*^i*π*/2^ and *γʹ* = −sin(*φ*′) + cos(*φ*′)*e*^i*π*/2^, respectively. For the 800-nm (400-nm) light fields, the spatial intensity distribution of the incident field is taken to be *D* = 1 when (*x*′^2^ + *y*′^2^)^1/2^ < 9 mm (4.5 mm), and *D* = 0 when (*x*′^2^ + *y*′^2^)^1/2^ > 9 mm (4.5 mm). During the numerical calculation, the 20th order Gauss-Legendre integral formula is used.

### CTMC simulation

We calculated the photoelectron momentum distributions of spatially sculpted fields using the CTMC model. In the model, the positions where the photoelectron tunnels are derived from the Laudau effective potential theory^48^, given by *I*_*p*_/|*E*(*t*)|, where |*E*(*t*)| is the instantaneous electric field strength and *I*_*p*_ is the ionization potential. The tunneled electron wavepackets have a Gaussian distribution on the transversal momentum (*v*_⊥_) perpendicular to the instantaneous direction of the synthesized two-color light field, expressed as: $\chi (v_ \bot ) = \sqrt {2I_p} /E(t) \cdot \exp [ - \sqrt {2I_p} (v_ \bot )^2/\left| {E(t)} \right|]$. And the ionized electrons have zero longitudinal momentum along the instantaneous laser field. Each electron trajectory is weighted by the ADK ionization rate^38^, $W_0(t) = \left| {(2I_p)^2/E(t)} \right|^{2/\sqrt {2I_p} - 1}\exp [ - 2(2I_p)^{3/2}/\left| {3E(t)} \right|]$, which determines the ionization rate with respect to the ionization instant. The ionization instants of electrons are sampled by the Monte Carlo method. After tunneling, the classical motion of electron outside the barrier is governed by the Newtonian equation, ${{{\ddot{\mathbf r}}}} = - {{{\mathbf{r}}}}/r^3 - {{{\mathbf{E}}}}(t)$, where *r* is the distance between electron and nucleus. The asymptotic momenta of the tunneled electrons on the virtual detector are transformed from the electron momenta when the laser is turned off according to the Kepler’s laws. After that, the electrons with positive energies are selected and their positions and momenta are recorded.

To simulate the photoelectron ionization dynamics in a spatially sculpted light field, the spatial intensity and phase distribution of the focal light field are included in the CTMC model. We calculate the light field near focus by employing the Richards–Wolf vectorial diffraction method^36^. For the calculation of the electric field at the focal plane, a 200 × 200 calculation grid with a bin size of Δ*x* = 0.4 μm was used. Within each calculation grid, the electric field was treated as a plane wave with a unified polarization state. The total momentum distribution of the electrons is given by the superposition of photoelectron momentum distributions ionized from different focal light field calculation grids. In our simulation, the two-color co-rotating circular field was synthesized by an eight 800-nm-cycle trapezoidal laser pulse, that was composed of a one-cycle ramp-on, a seven-cycle plateau and a one-cycle ramp-off. For the final momentum distributions, a 300 × 300 calculation grid with a bin size of Δ*p* = 0.01a.u. was selected.

### Analytical derivation of angle-resolved photoelectron yield

Considering the intensity and phase profiles in transverse plane, the electric field of the driving laser field can be written as: **E**(*t*,*φ*) = *E*_pr_*g*_pr_(*φ*)[cos(*ω*_pr_*t*)**e**_*x*_ − $\left. {\sin \left( {\omega _{{{{\mathrm{pr}}}}}t} \right){{{\mathbf{e}}}}_y} \right] + E_{{{{\mathrm{un}}}}}g_{{{{\mathrm{un}}}}}(\varphi )[\cos (\omega _{{{{\mathrm{un}}}}}t + \Delta \phi ){{{\mathbf{e}}}}_x - \sin (\omega _{{{{\mathrm{un}}}}}t + \Delta \phi ){{{\mathbf{e}}}}_y].$ Likewise, the electric vector potential of the driving laser field can be expressed as: ${{{\mathbf{A}}}}(t,\varphi ) = - {\textstyle{{E_{{{{\mathrm{pr}}}}}} \over {\omega _{{{{\mathrm{pr}}}}}}}}g_{{{{\mathrm{pr}}}}}(\varphi )$ $[\sin (\omega _{{{{\mathrm{pr}}}}}t){{{\mathbf{e}}}}_x + \cos (\omega _{{{{\mathrm{pr}}}}}t){{{\mathbf{e}}}}_y] - {\textstyle{{E_{{{{\mathrm{un}}}}}} \over {\omega _{{{{\mathrm{un}}}}}}}}g_{{{{\mathrm{un}}}}}(\varphi )[\sin (\omega _{{{{\mathrm{un}}}}}t + \Delta \phi ){{{\mathbf{e}}}}_x + \cos (\omega _{{{{\mathrm{un}}}}}t + \Delta \phi ){{{\mathbf{e}}}}_y].$ Here, the notations are the same as that described in the main text. Since the OAM of vortex pulses is related to the azimuthal change of phase, here, the radial variation of the two-color field can be neglected. In general, the final drift momentums of photoelectrons mostly distribute near the negative vector potential^49^, having the classical relation **p** ~ −**A**(*t*_0_,*φ*). Here *t*_0_ is the ionization instant and the electron emitting angle is given by *ξ* = arctan(*A*_*y*_/_*Ax*_). If the peak electric field strength of the unknown beam is much weaker than the probing beam, the influence of unknown light field in deriving the emitting angle can be neglected and one can obtain the concise mapping relation *ξ* = *ω*_pr_*t*_0_. Inserting this relationship and the electric field expression **E**(*t*,*φ*) into the Ammosov–Delone–Krainov (ADK) ionization rate formula^37^, we can express the angle-resolved photoelectron yield *W* as a function of the electron emitting angle *ξ*, and the azimuthal location *φ*:

4 $$W(\xi ,\varphi ) = [(2I_p)^2/\left| {E(\xi ,\varphi )} \right|]^{2/\sqrt {2I_p} - 1} \cdot \exp \left( - \frac{{2(2I_p)^{3/2}}}{{3\left| {E(\xi ,\varphi )} \right|}}\right)$$

i.e., Eq. (1) in the main text. Here, the photoelectron yield is positively correlated with the electric field strength $\left| E \right| = \sqrt {\varepsilon _{{{{\mathrm{pr}}}}}^2g_{{{{\mathrm{pr}}}}}^2 + \varepsilon _{{{{\mathrm{un}}}}}^2g_{{{{\mathrm{un}}}}}^2 + 2\varepsilon _{{{{\mathrm{pr}}}}}\varepsilon _{{{{\mathrm{un}}}}}g_{{{{\mathrm{pr}}}}}g_{{{{\mathrm{un}}}}}\cos (\xi + \Delta \phi )}$, in which the ionization instant has been replaced by *t*_0_ = *ξ*/*ω*_pr_. Equation (4) establishes a correlation between the electron emitting angle and the corresponding photoelectron yield, which can be directly associated with the measured PMDs by using COLTRIMS. One should note that Eq. (4) describes the photoelectron yield from a single position of the light field. For each azimuthal angle *φ*, the local photoelectron yield *W* reaches a peak when the electron emitting angle satisfies *ξ* = −Δ*ϕ*. This peak corresponds to a maximum in PMD, leading to the typical crescent-shaped lobe pattern^37^.

In this work, the spatial effect of the driving laser field has to be considered. The measured PMDs are the superposition of photoionization evens generated from the whole focal plane. Employing Eq. (4), the experiment results in the main text can be well explained. When the probing light field has not been spatially sculptured (Fig. 2a–c), the intensity of the two-color laser field is cylindrical symmetrical. The total angle-resolved photoelectron yield can be given by

5 $$F(\xi ) = {\int}_0^{2\pi } {W(\xi ,\varphi )} d\varphi$$

When the OAM of the unknown light field is zero, the relative phase of the two-color field is independent of the azimuthal location. In this case, Eq. (5) can be simplified as *F*(*ξ*) = 2*πW*(*ξ*,0). The total angle-resolved photoelectron yield is linearly related with *W*. The typical crescent-shaped lobe pattern is therefore observed in Fig. 2a. When the OAM value of unknown pulses is non-zero, according to the expression of |*E*(*t*)|, we can obtain the relation *W*(*ξ*,*φ*) = *W*(0,*φ* + *ξ*/*ℓ*_400_). Besides, since the photoelectron yields *W* is a periodic function of the azimuthal angle *φ* (namely, *W*(*ξ*,*φ*) = *W*(*ξ*,*φ* + 2*π*)), we have ${\int}_0^{2\pi } {W(\xi ,\varphi )} d\varphi = {\int}_0^{2\pi } {W(\xi ,\varphi + a)} d\varphi$, in which *a* is an arbitrary real number. Consequently, we will have *F*(0) = *F*(*ξ*). This indicates that the total photoelectron yield *F* is actually not a function of the electron emitting angle. So, the PMDs in Fig. 2b, c manifest as an annulus.

On the other hand, when the probing light field is spatially sculptured by the slit (Fig. 3), the spatial profile *g* can be approximated as *g*(*φ*) = *δ*(*φ* *−* *φ*_s_) + *δ*(*φ* *−* *φ*_s_ − *π*), in which *δ* has been defined in the main text. Inserting this formula into Eq. (5), the total angle-resolved yield can be written as: *F*(*ξ*) = *W*_1_(*ξ,* *−* *π/2*) + *W*_2_(*ξ,π/2*). When the topological charge of the unknown light field is *ℓ* = 1, *W*_1_ reaches its maximum value when *W*_2_ reaches its minimum value. Thus, the total PMD is similar to an annulus (Fig. 4a). Likewise, when the topological charge of the unknown light field is *ℓ* = 2, the total angle-resolved yield can be simplified as *F*(*ξ*) = 2 *W*(*ξ,π*), which leads to the typical crescent-shaped lobe pattern as shown in Fig. 4b.

## Supplementary information

Supplementary Information

## Data Availability

The datasets generated and analyzed during the current study are available from the corresponding author on reasonable request.
