# Supplementary material for: Probing the orbital angular momentum of intense vortex pulses with strong-field ionization
Source: Light Sci Appl. 2022 Feb 8;11:34. doi: 10.1038/s41377-022-00726-7 (PMC8821541; doi:10.1038/s41377-022-00726-7)
Supplement: Supplementary file 1 — Supplementary Information [file 41377_2022_726_MOESM1_ESM.docx]

**Supplementary Information for:**

**Probing the orbital angular momentum of intense vortex pulses with strong-field ionization**

Yiqi Fang,^1^ Zhenning Guo,^1^ Peipei Ge,^1^ Yankun Dou,^1^ Yongkai Deng,^1^ Qihuang Gong,^1,2,3,4^ & Yunquan Liu^1,2,3,4,*^

^1^ State Key Laboratory for Mesoscopic Physics and Frontiers Science Center for Nano-optoelectronics, School of Physics, Peking University, Beijing 100871, China

^2^Collaborative Innovation Center of Extreme Optics, Shanxi University, Taiyuan, Shanxi 030006, China

^3^Center for Applied Physics and Technology, HEDPS, Peking University, Beijing 100871, China

^4^ Beijing Academy of Quantum Information Sciences, Beijing 100193, China

* Correspondence to: (Y.L.) Yunquan.liu@pku.edu.cn

1. **Relationship between the photoelectron momentum distributions and the chirality of SAM and OAM of probing fields.**

Here, we further investigate the more general cases in which the effect of the SAM and OAM of the 800-nm probing fields is comprehensively studied. We consider the below cases: (i) *ℓ*_800_ = 0 and *σ*_800_ = *σ*_400_, where *ℓ*_800_ is the topological charge of the probing 800-nm field and *σ*_800_ and *σ*_400_ are the spin state of the 800-nm field and 400-nm field respectively; (ii) *ℓ*_800_ = 0 and *σ*_800_ = -*σ*_400_; (iii) *ℓ*_800_ = 1 and *σ*_800_ = *σ*_400_; (iv) *ℓ*_800_ = 1 and *σ*_800_ = -*σ*_400_; (v) *ℓ*_800_ = -1 and *σ*_800_ = *σ*_400_; and (vi) *ℓ*_800_ = -1 and *σ*_800_ = -*σ*_400_.

The simulated results of (i) and (ii) are shown in Fig. S1, and the results of (iii)-(vi) are exhibited in Fig. S2. As shown in Fig. S1, if the SAM state of the probing field is the same as that of the 400-nm vortex fields, one can characterize the OAM states of optical vortices by a crescent-shaped lobe. Likewise, if the SAM states of these two fields are opposite, the OAM state of 400-nm vortices can be monitored by a three-lobe shape. As shown in Fig. S2, when the 800-nm probing field carries OAM whose sign is the same as that of 400-nm field, the electron momentum distributions are determined by the OAM of 400-nm vortices. If the sign of 800-nm field OAM is opposite to the 400-nm vortices, the electron momentum distributions reveal annulus structures. The simulated results indicate that the chirality of probing beams’ SAM and OAM provides a flexible toolbox for the detection of optical vortices’ OAM state.


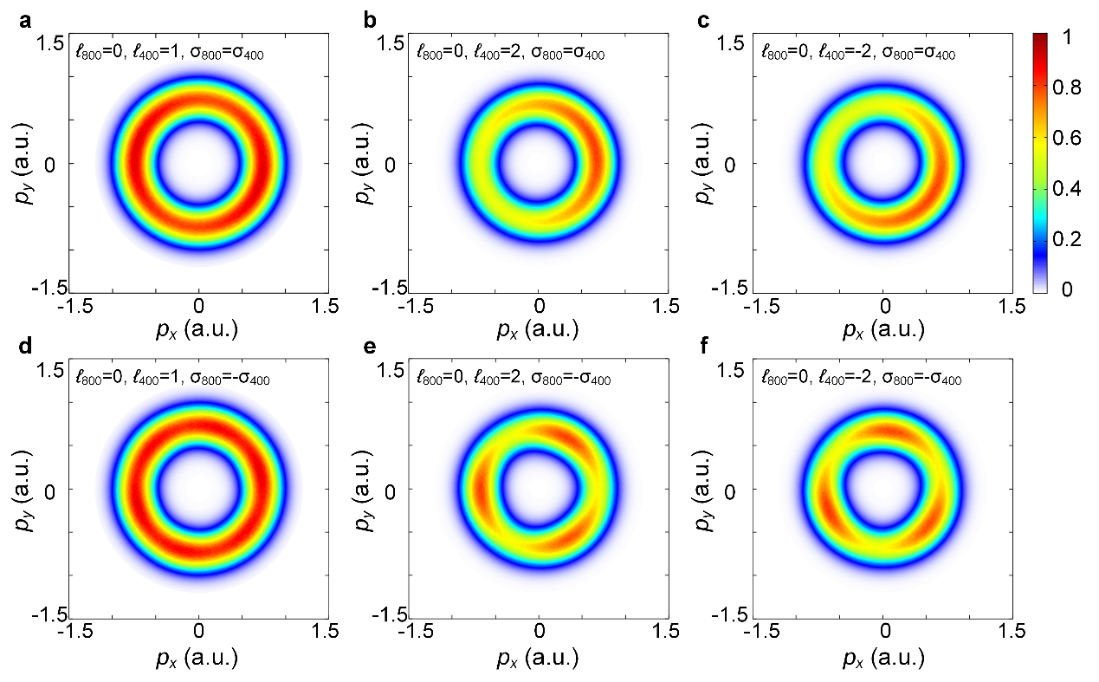


**Fig. S1. Simulated photoelectron momentum distributions with probing fields of zero OAM.** The probing fields are structured by the 2-mm slit. **a-c** The spin state of the probing fields is the same as the optical vortices which need to be measured. **d-f** The spin state of the probing fields is opposite to the optical vortices.


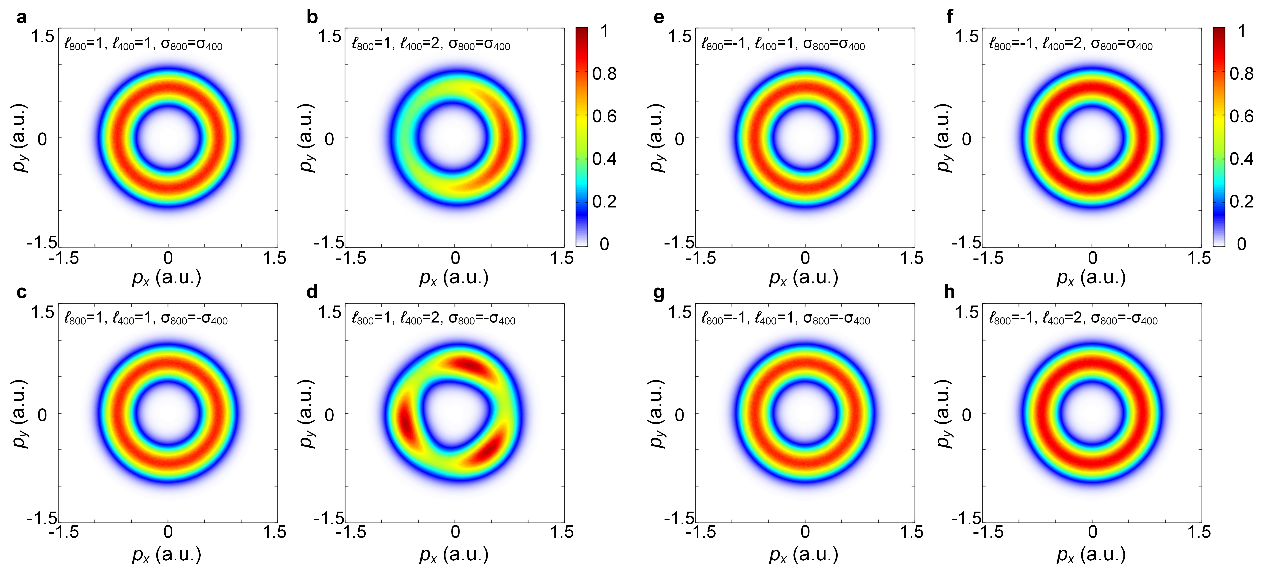


**Fig. S2. Simulated photoelectron momentum distributions with probing fields of non-zero OAM.** **a-d** Simulated results with positive-OAM probing fields. **e-h** Simulated results with negative-OAM probing fields. In **a**, **b**, **e** and **f**, the spin state of the probing fields is the same as the optical vortices which need to be measured. In **c**, **d**, **g** and **h**, the spin state of the probing fields is opposite to the optical vortices.
